# Vaccarin suppresses diabetic nephropathy through inhibiting the EGFR/ERK1/2 signaling pathway: Vaccarin ameliorates diabetic nephropathy

**DOI:** 10.3724/abbs.2024141

**Published:** 2024-08-27

**Authors:** Xuexue Zhu, Xinyu Meng, Xinyao Du, Chenyang Zhao, Xinyu Ma, Yuanyuan Wen, Shijie Zhang, Bao Hou, Weiwei Cai, Bin Du, Zhijun Han, Fei Xu, Liying Qiu, Haijian Sun

**Affiliations:** 1 Department of Basic Medicine Wuxi School of Medicine Jiangnan University Wuxi 214122 China; 2 Department of Clinical Research Center Jiangnan University Medical Center Wuxi 214001 China; 3 State Key Laboratory of Natural Medicines China Pharmaceutical University Nanjing 210009 China

**Keywords:** diabetic nephropathy, vaccarin, EMT, renal fibrosis, EGFR

## Abstract

Diabetic nephropathy (DN) is recognized as one of the primary causes of chronic kidney disease and end-stage renal disease. Vaccarin (VAC) confers favorable effects on cardiovascular and metabolic diseases, including type 2 diabetes mellitus (T2DM). Nonetheless, the potential role and mechanism of VAC in the etiology of DN have yet to be completely elucidated. In this study, a classical mouse model of T2DM is experimentally induced via a high-fat diet (HFD)/streptozocin (STZ) regimen. Renal histological changes are assessed via H&E staining. Masson staining and immunohistochemistry (IHC) are employed to assess renal fibrosis. RT-PCR is utilized to quantify the mRNA levels of renal fibrosis, oxidative stress and inflammation markers. The levels of malondialdehyde (MDA) and reactive oxygen species (ROS), as well as the content of glutathione peroxidase (GSH-Px), are measured. The protein expressions of collagen I, TGF-β1, α-SMA, E-cadherin, Nrf2, catalase, SOD3, SOD2, SOD1, p-ERK, p-EGFR (Y845), p-EGFR (Y1173), p-NFκB P65, t-ERK, t-EGFR and t-NFκB P65 are detected by western blot analysis. Our results reveal that VAC has a beneficial effect on DN mice by improving renal function and mitigating histological damage. This is achieved through its inhibition of renal fibrosis, inflammatory cytokine overproduction, and ROS generation. Moreover, VAC treatment effectively suppresses the process of epithelial-mesenchymal transition (EMT), a crucial characteristic of renal fibrosis, in high glucose (HG)-induced HK-2 cells. Network pharmacology analysis and molecular docking identify epidermal growth factor receptor (EGFR) as a potential target for VAC. Amino acid site mutations reveal that Lys-879, Ile-918, and Ala-920 of EGFR may mediate the direct binding of VAC to EGFR. In support of these findings, VAC reduces the phosphorylation levels of both EGFR and its downstream mediator, extracellular signal-regulated kinase 1/2 (ERK1/2), in diabetic kidneys and HG-treated HK-2 cells. Notably, blocking either EGFR or ERK1/2 yields renal benefits similar to those observed with VAC treatment. Therefore, this study reveals that VAC attenuates renal damage via inactivation of the EGFR/ERK1/2 signaling axis in T2DM patients.

## Introduction

For several decades, diabetic nephropathy (DN) has been considered a leading cause of chronic kidney disease and end-stage renal disease [ 1, 2]. Recently, it has been estimated that 550 million people worldwide will suffer from diabetes by 2030, and approximately 50% of patients with type 2 diabetes will develop DN [ 3, 4]. DN is characterized by proteinuria, excessive mesangial matrix formation and renal fibrosis [ 5, 6]. Renal fibrosis is the main pathological event in the development of DN [7]. Renal fibrosis usually manifests as overproduction of the extracellular matrix (ECM), including collagen I, in the renal tubulointerstitium [8]. Renal tubular epithelial cells are damaged in the early stage of DN [9]. Long-term exposure to hyperglycemia leads to the transformation of tubular structure to renal interstitial structure, which is also known as epithelial-to-mesenchymal transition (EMT). Once the process of EMT occurs, the ECM is dispersed around tubular structures, causing irreversible damage to the renal structure [ 10, 11]. Accumulating evidence suggests that transforming growth factor-β1 (TGF-β1) plays a pivotal role in DN progression by triggering the TGF-β1/Smad signaling pathway, a significant fibrogenic pathway [ 12, 13] .

Epidermal growth factor receptor (EGFR) is generally expressed in renal epithelial cells [ 14, 15]. EGFR binding to ligands leads to activation of the intrinsic kinase domain at Y1173 [ 14, 16]. In addition, EGFR can be phosphorylated at Y845 via a nonligand pathway mediated by oxidative stress [17]. Mounting evidence indicates that EGFR is a pivotal mediator in the process of renal fibrosis [ 18, 19]. High glucose (HG)-mediated EGFR phosphorylation and ERK1/2 activation promote TGF-β expression and induce renal fibrosis [17]. Despite the increasing understanding of its pathology, DN remains a leading cause of mortality in diabetic patients. Therefore, the development of novel effective drugs to prevent or treat DN is imperative.

Vaccarin (VAC), a natural flavonoid glycoside [20], has many pharmacological effects, such as antioxidation, anti-inflammatory and antihyperglycemic effects [ 21– 23]. Our group has shown that VAC alleviates HG- and hydrogen peroxide (H _2_O _2_)-induced endothelial cell injury via inhibition of Notch signaling [ 24, 25]. In addition, VAC treatment ameliorates nephropathy and cardiovascular remodeling in hypertensive rats [26]. Recently, it has been demonstrated that VAC improves glucose and lipid metabolism disorders in type 2 diabetes mellitus (T2DM) mice [ 27 , 28]. Moreover, VAC prevents ox-LDL-induced injury in endothelial cells by suppressing the reactive oxygen species (ROS)/mitogen-activated protein kinase (MAPK) signaling pathway [20]. Overall, VAC may be beneficial for attenuating cardiovascular and metabolic disorders. However, little is known regarding the role and underlying mechanism of VAC in DN. Our network pharmacology analysis revealed that EGFR is a potential target of VAC. Whether and how EGFR mediates the renal benefits of VAC are largely unknown.

The present study explored the potential effects of VAC on DN and investigated whether VAC ameliorates renal injury by acting on the EGFR/ERK1/2 signaling pathway in T2DM mice.

## Materials and Methods

### Reagents

VAC ( Supplementary Figure S1) was purchased from Shifeng Technology (Shanghai, China). D-glucose was purchased from Sigma (St Louis, USA). Insulin was obtained from Solarbio (Beijing, China). Kits for serum creatinine (Scr), blood urea nitrogen (BUN) and urine protein were procured from Jiancheng Bioengineering Institute (Nanjing, China). Mouse INS (Insulin) ELISA Kit was purchased from Elabscience Biotechnology (Wuhan, China). Antibodies against β-actin, α-SMA, E-cadherin and p-ERK were obtained from Cell Signaling Technology (Danvers, USA). Antibodies against collagen I, TGF-β1, t-EGFR, p-EGFR (Y845), p-EGFR (Y1173) and t-ERK were procured from Abcam (Cambridge, USA). Primary antibodies against Nrf2, p-NFκB P65 and t-NFκB P65 were purchased from Proteintech (Wuhan, China). Primary antibodies against catalase, SOD1, SOD2 and SOD3 were procured from Boster Biological Technology (Wuhan, China).

### Experimental animals

Male C57BL/6J mice, aged 6–8 weeks, were obtained from the Model Animal Research Center of Nanjing University (Nanjing, China). All experiments were approved by the Institutional Animals Care and Use Committee at Jiangnan University (document number for animal use approval: JN.No20200710c0600131 [174]). The animals were housed in a controlled environment with a 12-h light-dark cycle, regulated temperature, and humidity. They were provided with unrestricted access to both standard chow and tap water.

### Mouse model establishment

The T2DM model was experimentally induced in mice via a high-fat diet (HFD)/streptozocin (STZ) regimen as previously reported [ 20, 27]. The normal control group (Ctrl) was given a standard diet. The group that received VAC daily (1 mg/kg, i.p.) for 8 weeks served as the VAC control group (VAC), whereas the other mice were fed with HFD (21.8 kJ/g, 60% fat, D12492). After being fed for 4 weeks, the HFD mice received a single dose of STZ intraperitoneally (120 mg/kg, pH 4.0, dissolved in 10 mM citrate buffer). Mice with fasting plasma glucose levels higher than 11.1 mM are diabetic [29]. Thereafter, T2DM mice were randomly allocated into two groups: the model group (DN) and the VAC-treated DN group (DN+VAC). The DN+VAC group was given VAC (1 mg/kg, i.p.) every day for 8 weeks. The mice in the DN group and DN+VAC group were maintained on a HFD until sacrifice.

### Oral glucose tolerance test (OGTT) and insulin tolerance test (ITT)

For the OGTT, all the animals were fasted overnight (12 h) and then orally administered with a single dose of D-glucose (2 g/kg). After one day of recovery, the animals were fasted for 4 h and intraperitoneally administered with insulin (0.75 units/kg, i.p.) to detect insulin sensitivity. The glucose levels were recorded with an AccuChek glucose meter at 0, 30, 60, 90, and 120 min after intragastric administration of glucose or intraperitoneal injection of insulin.

### Assessment of blood glucose levels, albuminuria, blood urea nitrogen and serum creatinine

Fasting blood glucose was measured via an AccuChek glucose meter at the end of the experiments. Then, metabolic cages were used to collect 24-h urine samples for albuminuria analysis. Blood samples were obtained to extract serum, and the serum concentrations of blood urea nitrogen (BUN) and creatinine (Scr) were assessed using the corresponding kits following the guidelines provided by the manufacturer [30].

### Measurement of plasma insulin

The levels of plasma insulin were determined via a mouse INS (Insulin) ELISA Kit according to the manufacturer’s instructions. The microwell plates were read with a microplate reader (BioTek, Winooski, USA).

### Sample collection and morphological observations

The kidneys were collected and weighed for renal/body weight index calculation. Kidneys were fixed with 4% paraformaldehyde, and kidney sections were cut at a thickness of 5 μm. Renal histological changes were assessed by both hematoxylin and eosin (H&E) staining and periodic acid-Schiff (PAS) staining. Masson staining was used to evaluate renal fibrosis. Images were captured with a Pannoramic SCAN (3DHISTECH, Budapest, Hungary).

### Immunohistochemistry (IHC)

Renal sections were deparaffinized, hydrated and subjected to antigen retrieval. The sections were then incubated with hydrogen peroxide (3%) to eliminate endogenous peroxidase activity, after which they were blocked with 5% BSA for 60 min. Primary antibodies against collagen I, α-SMA, and E-cadherin were then used for incubation overnight at 4°C. The sections were subsequently exposed to secondary antibodies coupled with horseradish peroxidase (HRP) for 60 min at room temperature. Finally, the sections were visualized with 3,3′-diaminobenzidine (DAB). The images were examined via the Pannoramic SCAN system.

### Quantitative real time-PCR (RT-PCR)

Total RNA was extracted from tissues or cells using TRIzol reagent (CWBIO, Beijing, China) according to the provided guidelines. An equal amount of RNA was subsequently subjected to reverse transcription using the HiScriptQ RT SuperMix (Vazyme, Nanjing, China), followed by quantitative real-time PCR using the ChamQTM SYBR®qPCR Master Mix (Vazyme). Relative gene expression levels were determined using the 2 ^‒ΔΔCT^ method [31]. The primer sequences are provided in Supplementary Table S1.

### Western blot analysis

Total protein was extracted using RIPA lysis buffer (CWBIO), and the protein concentration was determined using a BCA kit (Beyotime Biotechnology, Shanghai, China). Subsequently, 20 μg of protein was subject to 8%‒12% sodium dodecyl sulfate-polyacrylamide gel electrophoresis (SDS-PAGE) and then transferred to polyvinylidene fluoride (PVDF) membranes. Afterward, the membranes were blocked with defatted milk in Tris-buffered saline (TBS) containing 0.1% Tween-20 (TBST) for 1 h and then incubated overnight at 4°C with primary antibodies. Following wash with TBST, the membranes were incubated with HRP-conjugated secondary antibodies (1:2000 dilution; CWBIO). Finally, the blots were visualized via a chemiluminescence detection system (Millipore Darmstadt, Germany) and semiquantified using ImageJ (National Institutes of Health, Bethesda, USA).

### Cell culture

HK-2 cells were cultured in low-glucose DMEM (5.5 mM glucose; Gibco, Carlsbad, USA) supplemented with 10% FBS and 1% penicillin/streptomycin (Gibco) in a 5% CO _2_ incubator. The cells were treated with 5 μM VAC for 12 h before HG (35 mM) was added and cultured for 48 h in the subsequent experiments.

### Database analysis

Genes related to DN or diabetic kidney diseases were screened from the DrugBank database ( https://go.drugbank.com/) and the GeneCards database ( https://www.genecards.org). The predicted targets of VAC were identified from the PharmMapper database ( http://www.lilab-ecust.cn/pharmmapper/). The DAVID database ( https://david.ncifcrf.gov/) was used to predict biological processes (BP). High-confidence proteins of the protein-protein interaction (PPI) network were constructed via the STRING database ( https://string-db.org/) and visualized via Cytoscape.

### Molecular docking of EGFR and VAC

The 3D structure of EGFR was downloaded from the RCSB Protein Data Bank (PDB) ( http://www.rcsb.org/). The molecular structure of VAC was obtained from the PubChem Database ( https://pubchem.ncbi.nlm.nih.gov/). AutoDock ( http://autodock.scripps.edu/) was used to dock EGFR and VAC via network pharmacology. The binding energy was used as a docking score to assess the binding strength between VAC and EGFR.

### Construction of point mutation of EGFR

Wild-type (WT) and Gly719, Arg841, Asn842, Lys879, Ile918 and Ala920 mutant plasmids (Gly719A, Arg841A, Asn842A, Lys879A, Ile918A and Ala920G) of EGFR were constructed using site-directed mutagenesis. Total RNA from HK-2 cells was extracted and reverse-transcribed into cDNA. PCR products were purified and digested with *Fse*I and *Asc*I (NEB), and then inserted into the pcDNA3.1 plasmid vector. EGFR mutant plasmids were generated through PCR using Phusion High-Fidelity DNA Polymerase (NEB). The related primers are as follows: EGFR-WT, forward: 5′-gaggagatctcgcttgcagggattccgtc-3′, reverse: 5′-gacggaatccctgcaagcgagatctcctc-3′; EGFR-Gly719A, forward: 5′-cgaacgcaccggaggccagcactttgatc-3′, reverse: 5′-gatcaaagtgctggcctccggtgcgttcg-3′; EGFR-Arg841A, forward: 5′-tcaccagtacgttcgcggctgccaggtcgc-3′, reverse: 5′-gcgacctggcagccgcg aacgtactggtga-3′; EGFR-Asn842A, forward: 5′-tgttttcaccagtacggccct ggctgccaggtcg-3′, reverse: 5′-cgacctggcagccagggccgtactggtgaaaaca-3′; EGFR-Lys879A, forward: 5′-gattccaatgccatccacgcgataggcactttgcc tcc-3′, reverse: 5′-ggaggcaaagtgcctatcgcgtggatggcattggaatc-3′; EGFR-Ile918A, forward: 5′-cgctggcaggggctccgtcatatggcttggatcc-3′, reverse: 5′-ggatccaagccatatgacggagcccctgccagcg-3′; and EGFR-Ala920G, forward: 5′-ggagatctcgctgccagggattccgtc-3′, reverse: 5′-gacggaatccct ggcagcgagatctcc-3′

### Measurement of oxidative stress markers

Cellular superoxide anion production was assessed via dihydroethidium (DHE, 10 μM) or 2,7-dichlorofluorescein diacetate (DCFH-DA, 10 μM) staining in a dim environment for 30 min at 37°C. A fluorescence microscope (Axio Vert A1; Zeiss, Oberkochen, Germany) was used to capture the images. Additionally, malondialdehyde (MDA) levels and glutathione peroxidase (GSH-Px) activity were evaluated using the corresponding kits following the manufacturer’s instructions [32].

### Statistical analysis

Data are presented as the mean±SEM. The number of animals per group was 6, and the number of replicates for the molecular experiments was 4. Statistical analysis was performed with one-way analysis of variance (one-way ANOVA) using Prism 8 (GraphPad software, San Diego, USA). *P*<0.05 was considered statistically significant.

## Results

### VAC alleviates renal dysfunction in diabetic mice

**Figure 1:**
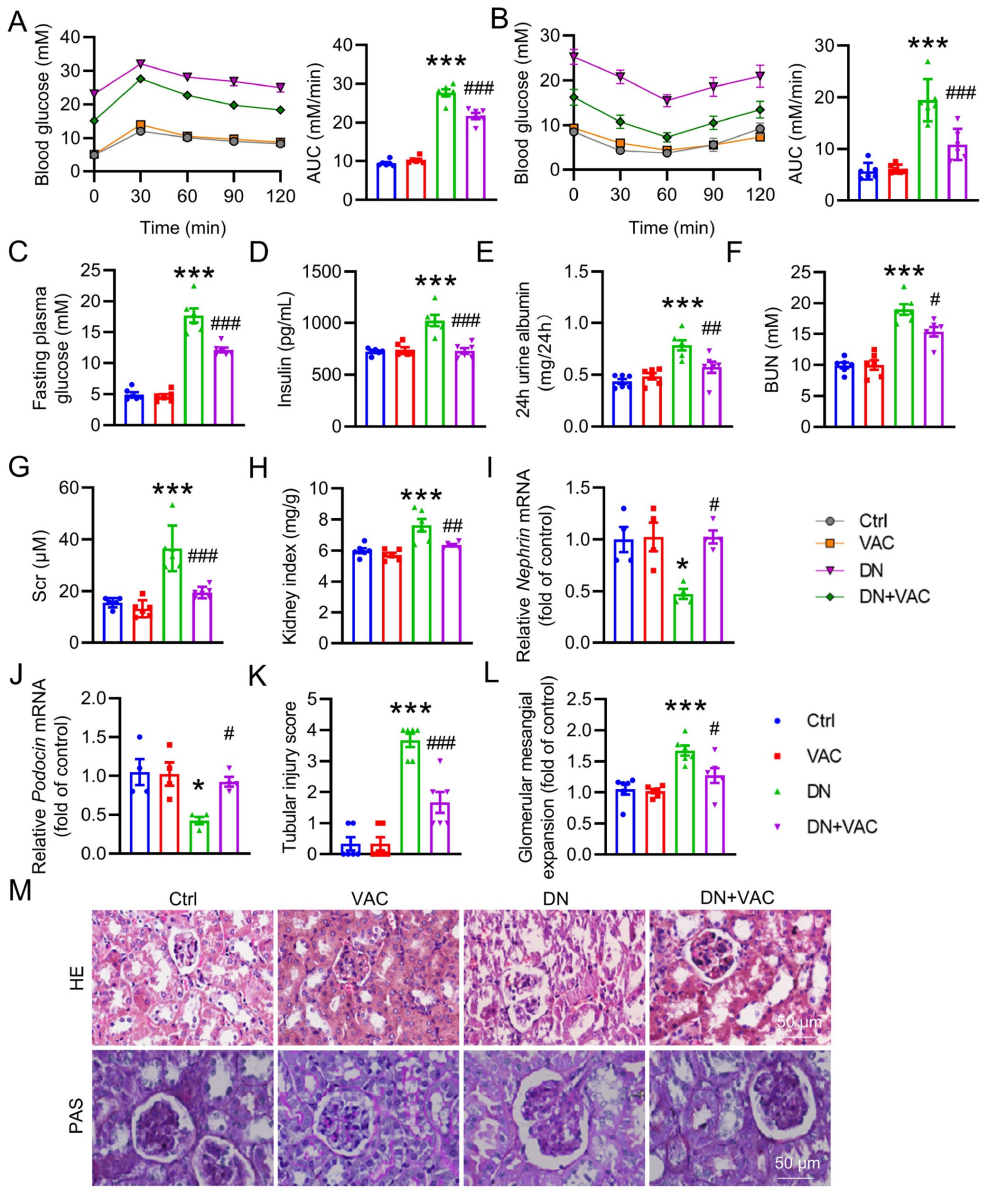
Figure 1 VAC alleviated renal function in T2DM mice (A) OGTT and AUC of the OGTT. n=6. (B) ITTs and AUCs. n=6. (C) Fasting blood glucose. n=6. (D) The level of plasma insulin. n=6. (E) The 24-h urine albumin analysis. n=6. (F) Serum BUN level. n=6. (G) Serum creatinine level. n=6. (H) Kidney/body weight ratio. n=6. (I,J) Relative mRNA levels of nephrin and podocin. n=4. (K) Tubular injury score. n=6. (L) Glomerular mesangial matrix expansion analysis. n=6. (M) Representative images of H&E and PAS staining. Scale bar: 50 μm. *P<0.05, ***P<0.001 vs Ctrl. #P<0.05, ##P <0.01, ###P<0.001 vs DN.

First, we assessed the therapeutic effects of VAC on glucose metabolism and renal damage induced by diabetes *in vivo*. As shown in Figure 1A,B, the mice in the DN group presented markedly impaired glucose tolerance and insulin sensitivity compared with the control group mice, and these effects were reversed after treatment with VAC. Compared with those in control mice, the fasting blood glucose and plasma insulin levels were significantly elevated in DN mice and reversed by VAC treatment ( Figure 1C,D). Moreover, the levels of urine ALB, BUN and Scr, as well as the renal/body weight index, were significantly greater in the DN group. However, treatment with VAC alleviated these effects in diabetic mice ( Figure 1E‒H). VAC supplementation noticeably restored the mRNA levels of *nephrin* and *podocin* in DN mice ( Figure 1I,J). Morphological analysis via H&E staining and PAS staining revealed that the glomeruli were hypertrophic in T2DM mice, which were obviously ameliorated by VAC ( Figure 1K‒M). Notably, there were no significant changes in renal morphology or renal function between normal mice and DN mice treated with VAC. These findings indicated that VAC improved glucose metabolic homeostasis and protected the kidneys from damage in T2DM mice.

### VAC attenuates renal fibrosis in diabetic mice

**Figure 2:**
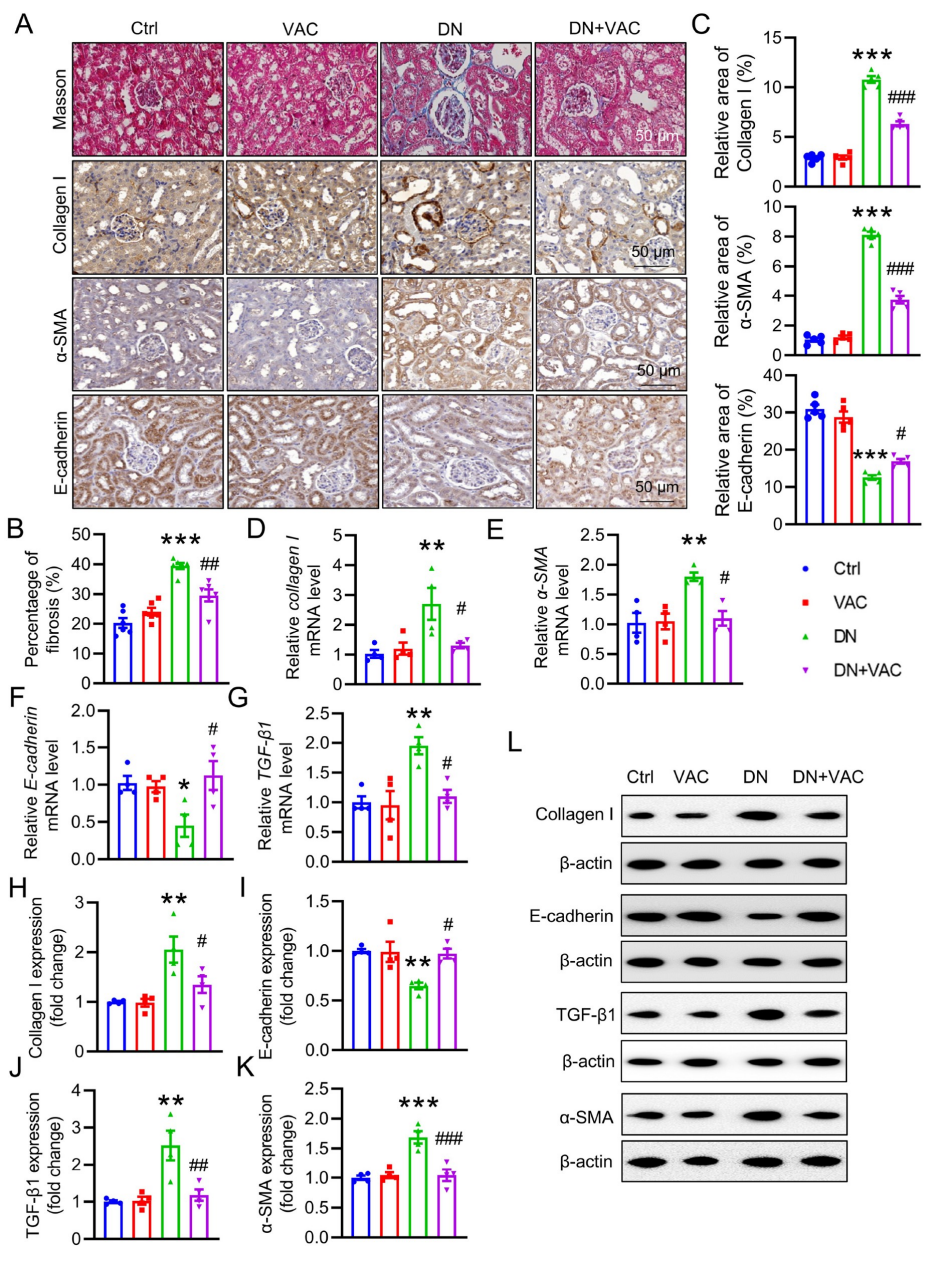
Figure 2 VAC alleviated renal fibrosis in T2DM mice (A) Images of kidney sections stained with Masson and subjected to immunohistochemistry for collagen I, α-SMA and E-cadherin in the mouse kidney. Scale bar: 50 μm. (B) Quantification of the fibrosis area via Masson’s trichrome staining. n=6. (C) Quantification of collagen I, α-SMA and E-cadherin expression. n=5. (D,E) Relative mRNA levels of collagen I, α-SMA, E-cadherin and TGF-β1. n=4. (H‒L) Representative blot images and quantitative analysis of collagen I, E-cadherin, TGF-β1 and α-SMA expressions. n=4. *P<0.05, **P<0.01, ***P<0.001 vs Ctrl. # P<0.05, ##P<0.01 vs DN.

Renal fibrosis is recognized as a fundamental priming stage in the pathogenesis of DN [33]. The accumulation of ECM is the primary feature of renal fibrosis [34]. Collagen I is one of the essential components of the ECM [35]. Masson staining revealed that VAC attenuated renal fibrosis in diabetic mice ( Figure 2A,B). The immunohistochemistry (IHC) results further confirmed that VAC reduced renal fibrosis in diabetic mice, as reflected by decreased collagen I immuno-positive signals ( Figure 2A,C). Similarly, increased *collagen I* mRNA levels were detected in DN mice compared with control mice. However, treatment with VAC substantially reversed these abnormalities ( Figure 2D). Abundant evidence underscores the involvement of EMT in the accumulation of ECM [ 36, 37 ]. This process involves an increase in the expressions of fibroblast markers, such as α-smooth muscle actin (α-SMA), and a reduction in the expressions of epithelial indicators, including E-cadherin [38]. IHC results indicated that VAC diminished the EMT process in T2DM mice, as indicated by lower α-SMA and higher E-cadherin immuno-positive signals ( Figure 2A,C). Consistent with the IHC results, VAC was found to downregulate the *α-SMA* mRNA level ( Figure 2E) and upregulate the *E-cadherin* mRNA level ( Figure 2F) in diabetic mice. TGF-β1 may play a key role in the process of EMT under diabetic conditions [ 39, 40 ]. RT-PCR results demonstrated that the upregulation of *TGF-β1* in T2DM mice was reduced by VAC treatment ( Figure 2G). In line with the RT-PCR results, the kidneys from DN mice presented increased protein expressions of collagen I, TGF-β1, and α-SMA but decreased protein expression of E-cadherin. Nevertheless, these effects were counteracted by the administration of VAC ( Figure 2H‒L). These data indicated that VAC alleviated renal fibrosis in the T2DM mice.

### VAC attenuates oxidative stress and inflammation in the kidney

**Figure 3:**
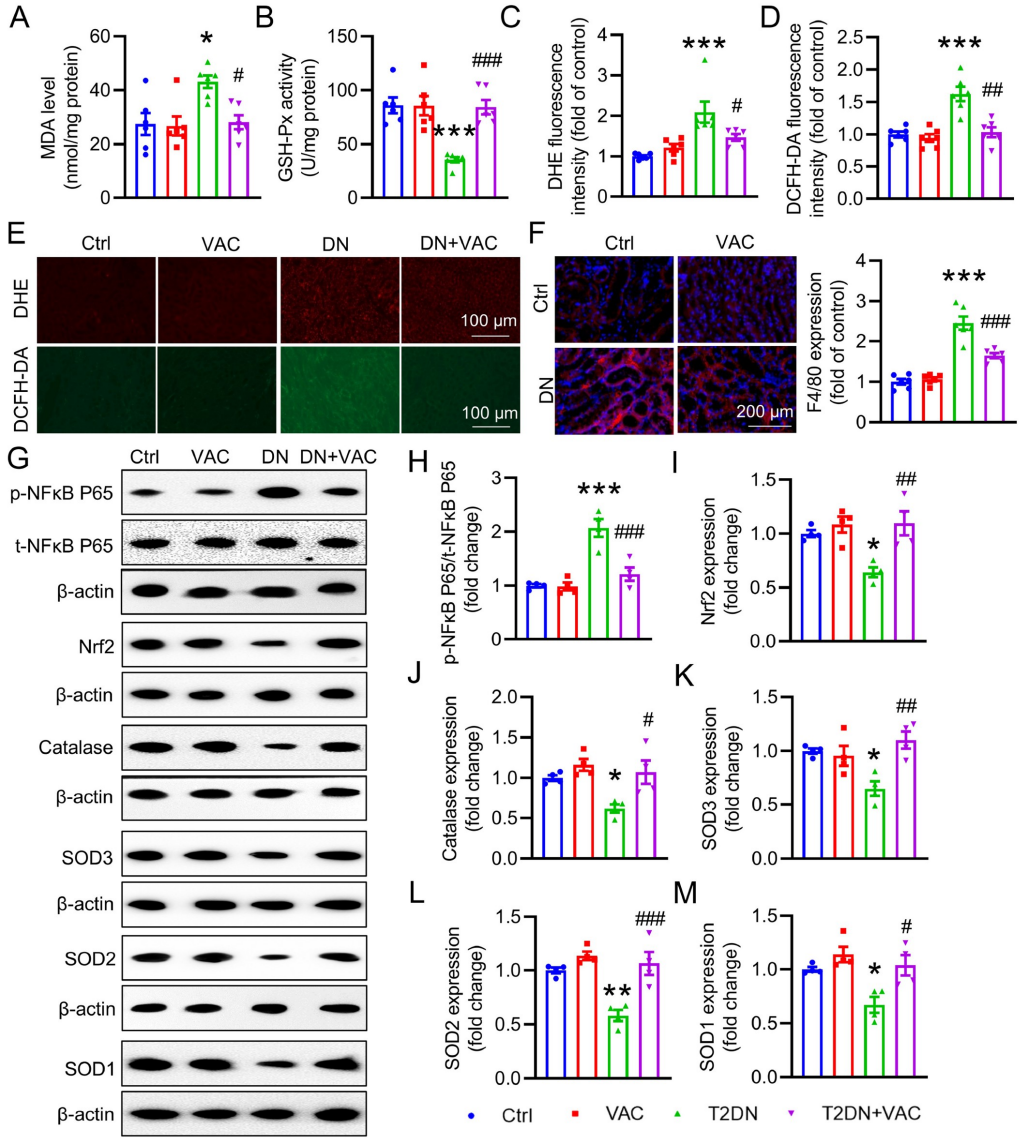
Figure 3 VAC alleviated the renal inflammatory response and oxidative stress in T2DM mice (A) MDA contents in diabetic kidney tissues. n=6. (B) GSH-Px activity in diabetic kidney tissues. n=6. (C) Averaged fluorescence intensity of DHE fluorescence in diabetic kidney tissues. n=6. (D) Averaged fluorescence intensity of DCFH-DA fluorescence staining of diabetic kidney tissues. n=6. (E) DHE fluorescence staining or DCFH-DA fluorescence staining of diabetic kidney tissues. Scale bar: 100 μm. (F) F4/80 staining of diabetic kidney tissues. Scale bar: 50 μm, and the average fluorescence intensity of F4/80-expressing diabetic kidney tissues is shown. (G‒M) Representative blot images and quantitative analysis of phosphorylated NFκB P65, Nrf2, catalase, SOD3, SOD2 and SOD1. n=4. *P<0.05, **P<0.01, ***P<0.001 vs Ctrl. #P<0.05, ##P <0.01, ###P<0.001 vs DN.

An abnormal inflammatory response and oxidative damage are the driving factors of DN [32]. Over the past decade, there has been significant interest in the role of Nrf2 in kidney biology, since Nrf2 protects against tubulointerstitial damage and reduces interstitial fibrosis in DN by inducing the expressions of several antioxidative genes, including heme oxygenase-1 ( *HO-1*), NADPH quinone oxidoreductase-1 ( *NQO-1*), glutamate-cysteine ligase synthetase catalytic ( *GCLC*) and glutamate-cysteine ligase synthetase modifier ( *GCLM*) [ 41, 42]. Hyperglycemia resulted in inflammatory responses in the renal system, as evidenced by increased mRNA levels of *IL-1β*, *VCAM-1* and *COX-2*, which were effectively reversed by VAC ( Supplementary Figure S2A–C). Additionally, we observed elevated MDA contents and reduced GSH-Px activities in the kidneys of diabetic mice. However, these anomalous alterations were mitigated by VAC treatment ( Figure 3A,B). Furthermore, DHE staining and DCFH-DA staining of renal tissues revealed diminished renal oxidative stress in diabetic mice treated with VAC ( Figure 3C‒E). Immunofluorescence staining demonstrated that the accumulation of macrophages was attenuated in DN mice treated with VAC ( Figure 3F). Similarly, the mRNA levels of *Nrf2*, *catalase*, *SOD3*, *SOD2*, and *SOD1*, as well as Nrf2-induced antioxidant genes, such as *HO-1*, *NQO-1* , *GCLC*, and *GCLM*, tended to be lower in DN mice, whereas VAC reversed these changes ( Supplementary Figure S2D‒H and Supplementary Figure S3A‒D). The western blot analysis results revealed that VAC reduced the phosphorylation level of NFκB P65 but elevated the protein expressions of Nrf2, catalase, SOD3, SOD2, and SOD1 in the renal samples of DN mice ( Figure 3G‒M). Collectively, these results suggested that VAC mitigated the inflammatory response and inhibited ROS production in diabetic kidneys.

### VAC attenuates HG-induced EMT in HK-2 cells

**Figure 4:**
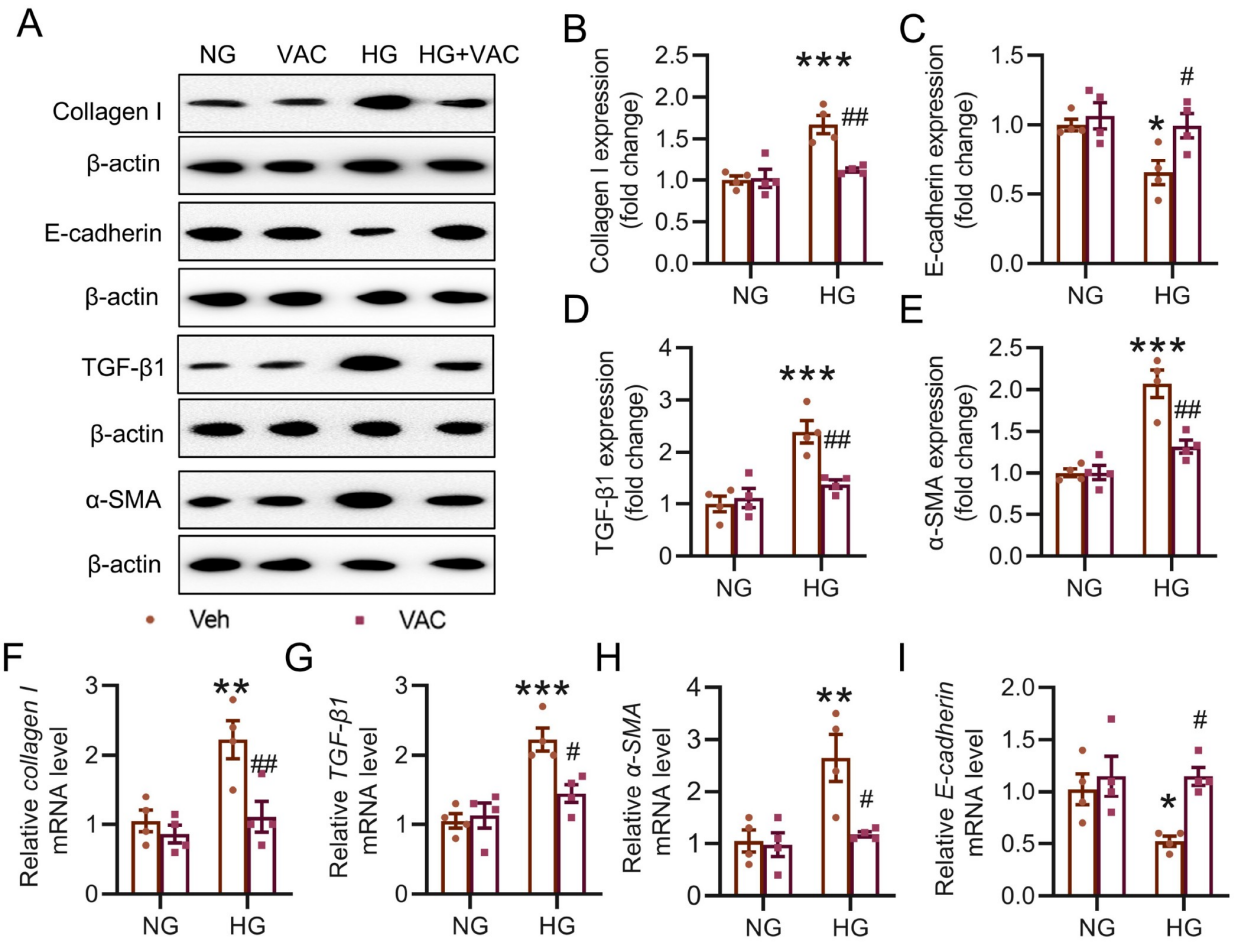
Figure 4 Effects of VAC on fibrosis in HK-2 cells (A–E) Representative blot images and quantitative analysis of collagen I, E-cadherin, TGF-β1 and α-SMA in HK-2 cells after 48 h of culture, both with and without VAC. (F–I) Relative mRNA levels of collagen I, TGF-β1, α-SMA and E-cadherin. *P<0.05, ** P<0.01, ***P<0.001 vs NG. #P <0.05, ##P<0.01 vs HG. n=4.

We extended our investigation to evaluate the potential impact of VAC on HG-induced HK-2 cells. Western blot analysis results demonstrated that pre-exposure to VAC effectively counteracted the increase in the protein levels of collagen I, α-SMA, and TGF-β1 but increased the decrease in the E-cadherin protein level in HG-exposed HK-2 cells ( Figure 4A‒E). Furthermore, as anticipated, VAC-treated cells exhibited no alteration in the progression of EMT, as indicated by the measurement of *collagen I* , *α-SMA*, *TGF-β1*, and *E-cadherin* mRNA levels ( Figure 4F‒I). These findings strongly suggested that the renoprotective effects of VAC may involve the suppression of the EMT process.

### VAC attenuates the HG-induced inflammatory response and oxidative injury in HK-2 cells

**Figure 5:**
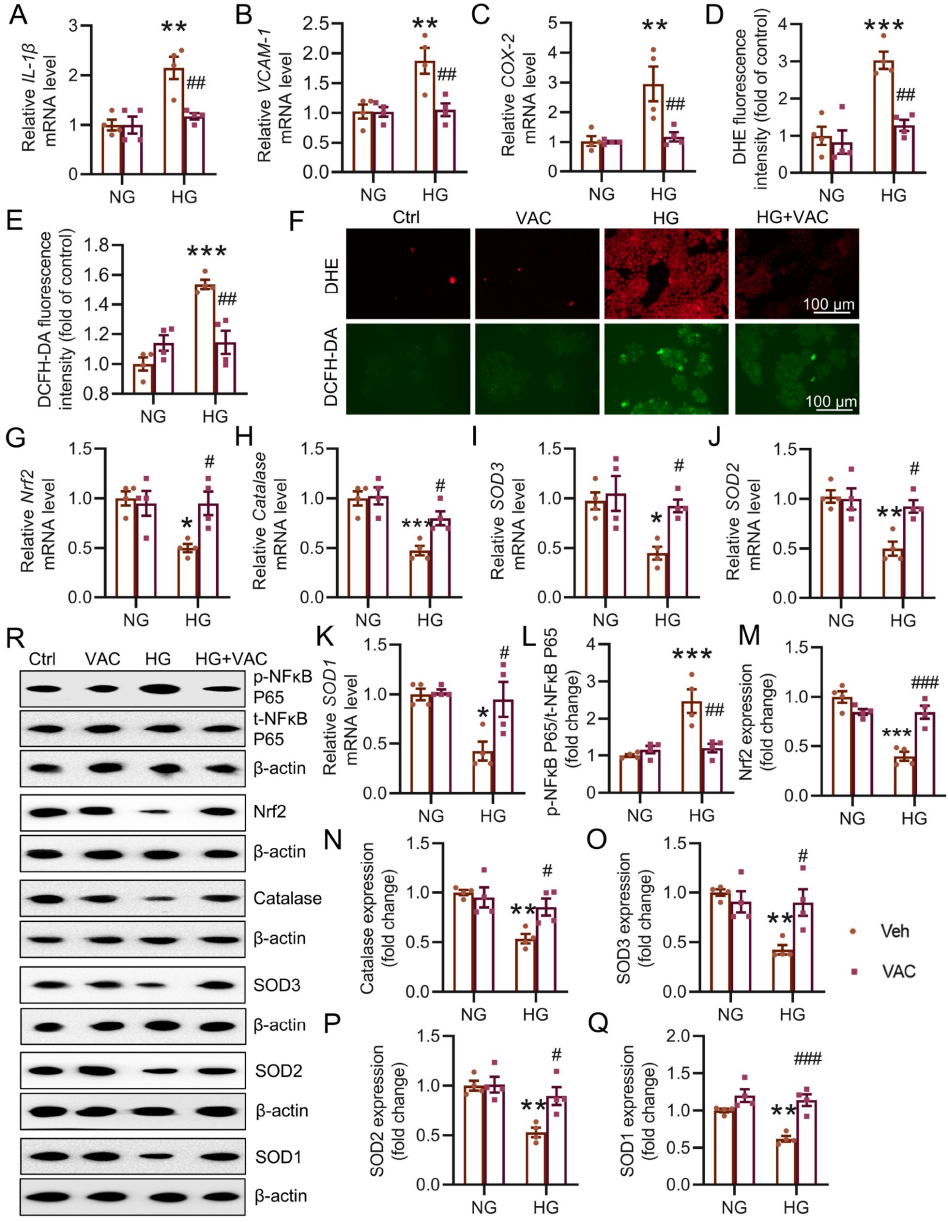
Figure 5 VAC alleviated the inflammatory response and oxidative stress in diabetic kidneys HK-2 cells were preincubated with 5 μM VAC for 12 h, followed by exposure to 35 mM HG for 48 h. (A–C) Relative mRNA levels of IL-1β , VCAM-1 and COX2. (D–F) DHE fluorescence staining or DCFH-DA fluorescence staining of HK-2 cells. Scale bar: 100 μm. (G–K) Relative mRNA levels of Nrf2, catalase, SOD3, SOD2 and SOD1. (L–R) Representative blot images and quantitative analysis of phosphorylated NFκB P65, Nrf2, catalase, SOD3, SOD2 and SOD1. *P<0.05, **P<0.01, ***P<0.001 vs NG. #P<0.05, ##P<0.01, ###P<0.001 vs HG. n=4.

We subsequently investigated whether VAC could alleviate HG-induced inflammation and oxidative stress in HK-2 cells. The mRNA levels of inflammatory marker genes, including *IL-1β*, *VCAM-1* and *COX-2*, significantly increased in response to HG exposure and were notably suppressed by VAC ( Figure 5A‒C). Furthermore, VAC preincubation effectively mitigated the excessive intracellular ROS generation triggered by HG in HK-2 cells ( Figure 5D–F). Consistent with the animal experiment results, VAC restored the mRNA and protein levels of *Nrf2*, *catalase*, *SOD3*, *SOD2*, and *SOD1*, as well as the downstream targets of Nrf2, including the Nrf2-dependent genes *HO-1* , *NQO-1*, *GCLC*, and *GCLM* in HK-2 cells upon exposure to HG ( Figure 5G‒K,M‒R and Supplementary Figure S4). Moreover, the increased phosphorylation of NFκB P65 in HG-exposed HK-2 cells was repressed in the presence of VAC ( Figure 5L,R). These data indicated that VAC inhibited the inflammatory response and ROS accumulation in HG-induced HK-2 cells.

### VAC ameliorates renal fibrosis by inhibiting the EGFR signaling pathway

**Figure 6:**
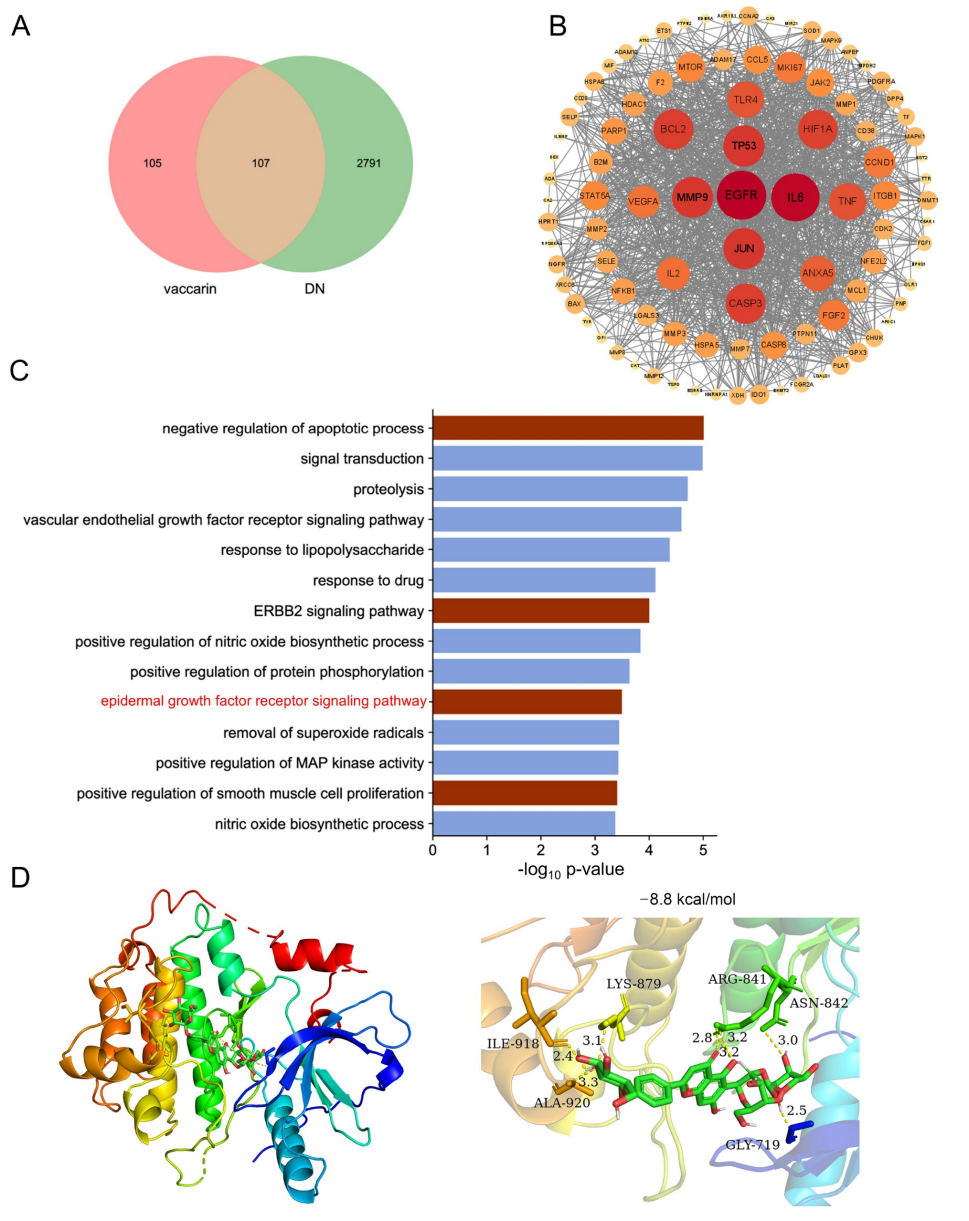
Figure 6 Network pharmacology analysis of the core targets of VAC and a molecular docking model of the core target with VAC (A) A Venn diagram depicting the overlap between candidates and interaction targets of VAC in the context of DN. (B) The PPI network of core targets was generated via the STRING database. (C) A bar graph illustrating the results of the biological process (BP) enrichment analysis. (D) Molecular docking of VAC with EGFR.

To further explore the underlying mechanism by which VAC alleviates renal fibrotic lesions in diabetic mice, we conducted network pharmacological analysis. A total of 107 genes associated with both VAC and DN were identified ( Figure 6A). A protein-protein interaction (PPI) network of common targets was constructed [43]. EGFR was obviously at the center of the network ( Figure 6B). Biological process (BP) is a significant aspect of GO (Gene Ontology) enrichment analysis [31]. We found that negative regulation of the apoptotic process, the ERBB2 signaling pathway and the epidermal growth factor receptor signaling pathway were significantly enriched ( Figure 6C) and were verified to contribute to renal fibrosis [ 34, 44 , 45]. Molecular docking demonstrated a direct interaction between VAC and EGFR, with a binding energy of –8.8 kcal/mol ( Figure 6D). The western blot analysis results revealed that the mutations in the Lys-879, Ile-918 and Ala-920 eliminated the inhibitory effects of VAC on ERK1/2 phosphorylation in HK-2 cells induced by hyperglycemia; this effect was not detected in Gly-719, Arg-841, or Asn-842 mutants ( Supplementary Figure S5). These results suggested that Lys-879, Ile-918, and Ala-920 might mediate the direct binding of VAC to EGFR.

**Figure 7:**
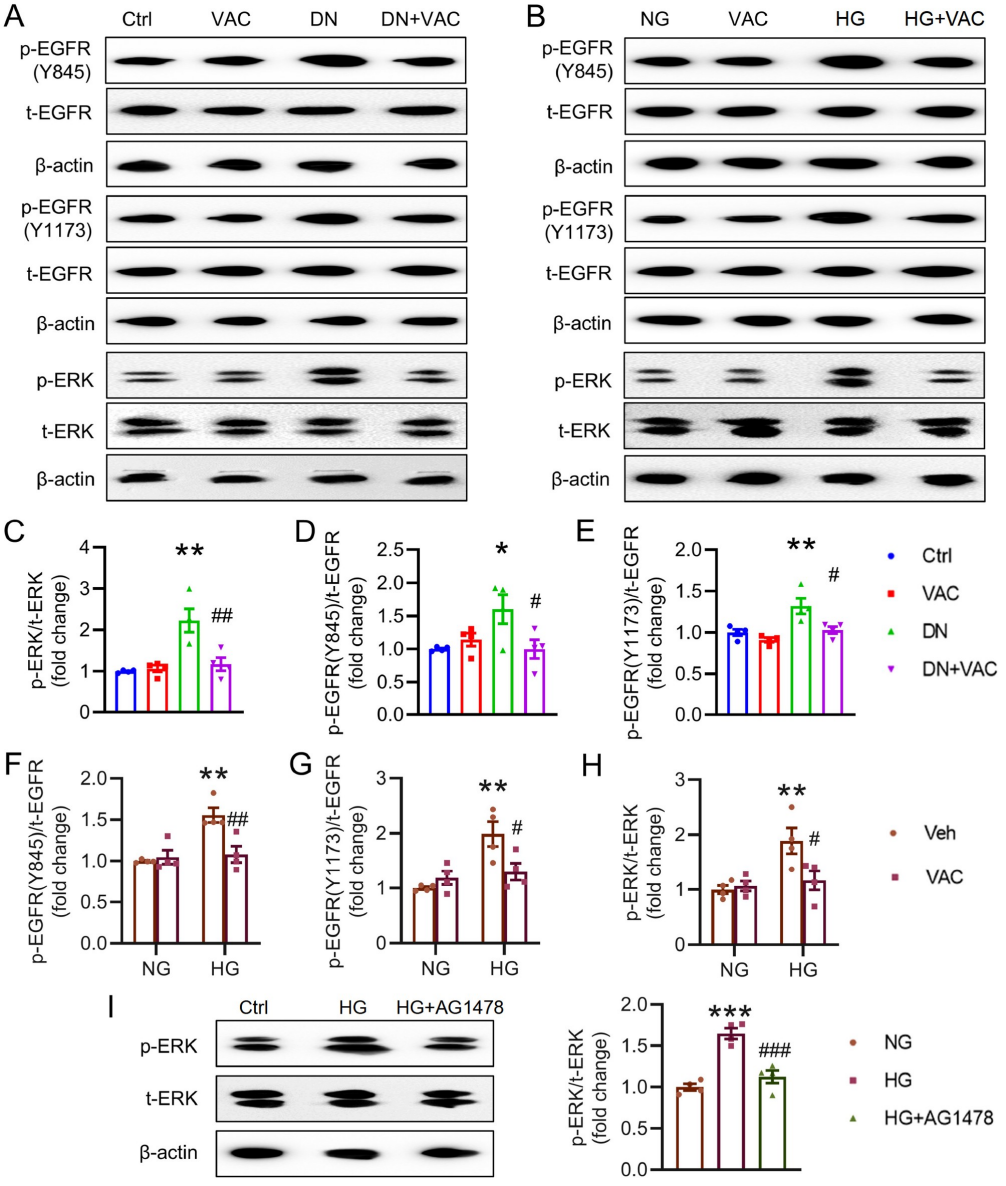
Figure 7 VAC inhibited EGFR/ERK phosphorylation in T2DM mice and HG-induced HK-2 cells (A) Representative blot images of p-EGFR and p-ERK in diabetic kidney tissues. (B) Representative blot images of HG-induced HK-2 cells. (C–E) Quantitative analysis of p-EGFR and p-ERK in diabetic kidney tissues. (F–H) Quantitative analysis of p-EGFR and p-ERK in HG-induced HK-2 cells. (I) Representative blot images and quantitative analysis of phosphorylated ERK1/2. *P<0.05, ** P<0.01, ***P<0.001 vs Ctrl or NG. # P<0.05, ##P<0.01, ### P<0.001 vs DN or HG. n=4.

EGFR is the possible key target of renal injury on the basis of bioinformatics and molecular docking. More importantly, EGFR activation is responsible for the development of DN through the activation of the ERK1/2 signaling pathway [15]. Therefore, we sought to determine whether VAC ameliorates renal injury by acting on the EGFR/ERK signaling pathway in T2DM patients. Our results revealed that the levels of phosphorylated EGFR (Y845, Y1173) and ERK1/2 in the kidney were increased, whereas this increase was abrogated by treatment with VAC ( Figure 7A,C‒E). Moreover, the HG-induced increase in EGFR/ERK phosphorylation in HK-2 cells was attenuated by VAC treatment, as demonstrated by western blot analysis ( Figure 7B,F‒H). Notably, the activation of ERK1/2 in HG-incubated cells was largely blocked by the EGFR inhibitor AG1478 ( Figure 7I), indicating that EGFR may be an upstream molecule that induces ERK1/2 phosphorylation in HK-2 cells.

**Figure 8:**
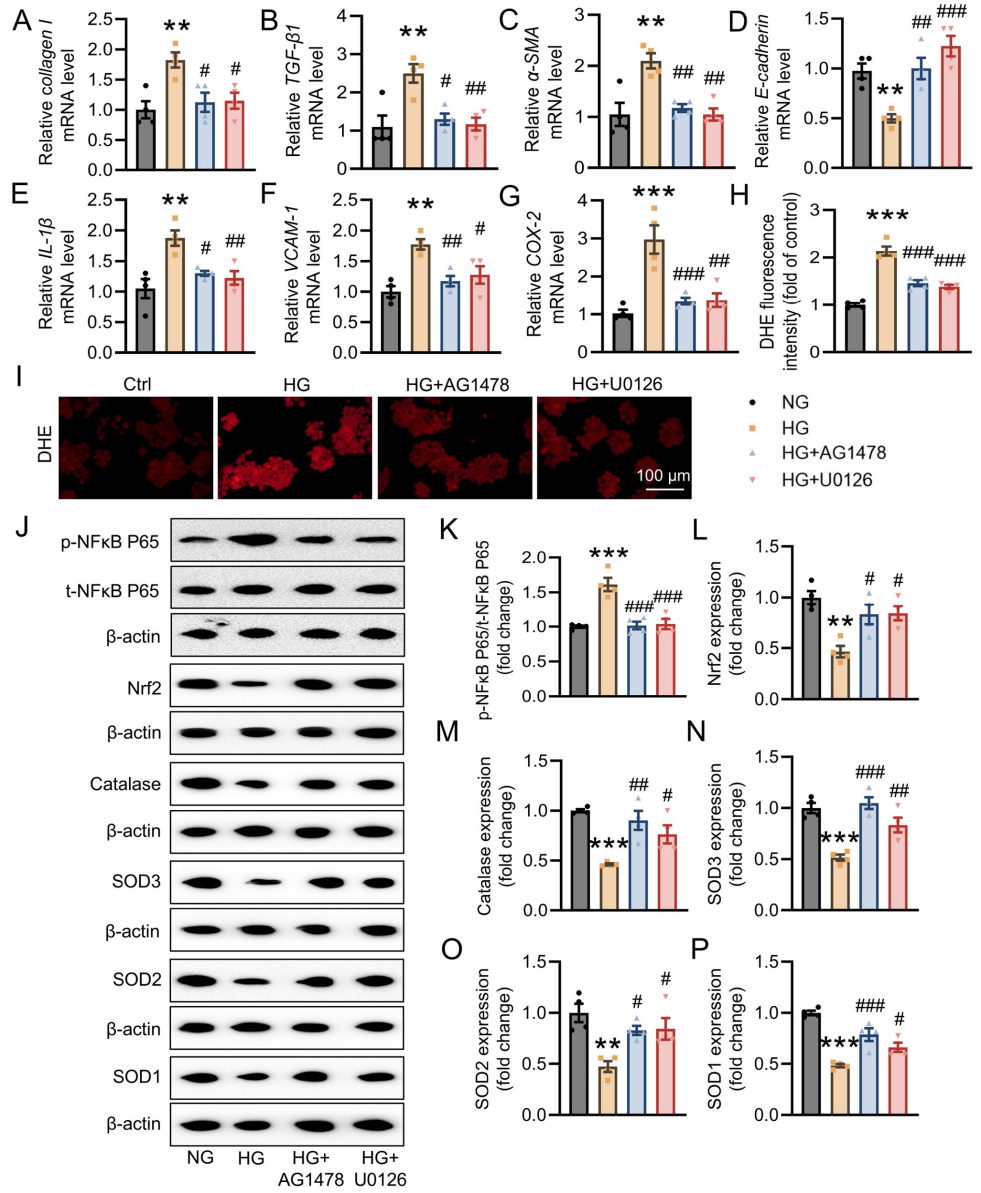
Figure 8 VAC ameliorated fibrosis, the inflammatory response and oxidative stress in HG-induced HK-2 cells exposed to the EGFR inhibitor AG1478 or the ERK inhibitor U0126 (A–D) Relative mRNA levels of collagen I, TGF-β1, α-SMA and E-cadherin in HK-2 cells. (E–G) Relative mRNA levels of IL-1β , VCAM-1 and COX-2 in HK-2 cells. (H) Averaged fluorescence intensity of DHE fluorescence in HK-2 cells. (I) DHE staining was performed on HG-induced HK-2 cells. Scale bar: 100 μm. (J–P) Representative blot images and quantitative analysis of phosphorylated and total NFκB P65, Nrf2, catalase, SOD3, SOD2 and SOD1. *P<0.05, ** P<0.01, ***P<0.001 vs NG. #P <0.05, ##P<0.01, ###P<0.001 vs HG. n=4.

Additional experiments were performed to test whether the EGFR/ERK signaling pathway contributes to hyperglycemia-induced renal damage; therefore, HK-2 cells were treated with or without the EGFR inhibitor AG1478 or the ERK inhibitor U0126 in the context of HG. We found that AG1478 or U0126 significantly suppressed HG-induced fibrosis ( Figure 8A‒D), inflammation ( Figure 8E‒G) and oxidative stress ( Figure 8H,I). Like pretreatment with VAC, pretreatment with either AG1478 or U0126 obviously weakened the HG-induced increase in phosphorylated NFκB P65 ( Figure 8J,K). Similarly, the diminished protein expression of Nrf2, catalase, SOD3, SOD2, and SOD1 in HG-stimulated cells was reversed by both AG1478 and U0126 ( Figure 8J,L‒P). These data indicated that VAC relieved hyperglycemia-induced renal injury by inactivating the EGFR/ERK1/2 signaling pathway.

## Discussion

In the present study, we found that VAC efficiently attenuated diabetic renal injury by preserving renal function, improving biochemical parameters, ameliorating morphological abnormalities and antagonizing renal fibrosis in mice. We also observed that VAC inhibited HG-induced EMT, inflammation and oxidative stress in HK-2 cells via inactivation of EGFR/ERK signaling. Our study suggested that VAC may be used as a new potential drug for the treatment of DN.

DN is a severe renal microvascular complication that arises as a consequence of persistent hyperglycemia [46]. Uncontrolled hyperglycemia causes renal structural damage, including glomerular lesions, microalbuminuria, mesangial expansion and interstitial fibrosis [ 4, 46]. In this study, VAC improved glucose intolerance and insulin resistance in diabetic mice. We also found that VAC improved diabetic renal dysfunction by reducing the levels of creatinine, BUN, and albuminuria. Histological examination further confirmed the therapeutic effects of VAC in DN.

Renal fibrosis plays an important role in the pathophysiology of DN [47]. Hyperglycemia leads to the EMT of renal tubular epithelial cells in DN, and EMT is necessary for myofibroblast activation and the synthesis of the ECM [48]. Our results showed that VAC retarded renal fibrotic processes in DN mice, as manifested by upregulated E-cadherin and downregulated collagen I, α-SMA and TGF-β1. These results indicated that VAC ameliorated the progression of renal fibrosis in diabetic mice by antagonizing the process of EMT. Inflammation and oxidative stress are believed to be the driving forces for the development of DN [49]. Nrf2 is a transcription factor that regulates genes responsible for cellular protection against oxidative stress and has anti-inflammatory functions in DN [ 50, 51]. In this study, we revealed that VAC exhibited an anti-inflammatory effect by inhibiting the release of inflammatory factors. Additionally, VAC alleviated oxidative stress in hyperglycemia-induced kidneys and HK-2 cells, as VAC enhanced the expressions of Nrf2 and its downstream targets, including HO-1, NQO-1, GCLC, and GCLM. These results demonstrated that VAC may be a promising agent for the treatment of DN by inhibiting the inflammatory response and ROS overproduction.

Using network pharmacology and molecular docking, we demonstrated that VAC might ameliorate renal injury by targeting EGFR. Compelling evidence indicates that EGFR plays a necessary role in the process of DN through the activation of the ERK1/2 signaling pathway [52]. Our data showed that VAC inhibited EGFR phosphorylation and ERK1/2 activation and that hyperglycemia-induced ERK1/2 phosphorylation was attenuated by the EGFR inhibitor AG1478. Moreover, blockade of either EGFR or ERK1/2 ameliorated the development of DN. Thus, VAC ameliorated renal fibrosis in DN mice through the inhibition of the EGFR-ERK signaling pathway. Further studies are needed to confirm whether overactivation of the EGFR/ERK signaling pathway counteracts the renoprotective effects of VAC in the setting of diabetes. Molecular docking revealed a direct interaction between VAC and EGFR, since VAC can bind to Gly-719, Arg-841, Asn-842, Lys-879, Ile-918, and Ala-920 of EGFR. Mutations of these binding sites revealed that only the Lys-879, Ile-918, and Ala-920 mutations eliminated the inhibitory effects of VAC on ERK1/2 phosphorylation in HK-2 cells induced by hyperglycemia; this effect was not detected in the Gly-719, Arg-841, and Asn-842 mutants. These results suggested that Lys-879, Ile-918, and Ala-920 might mediate the direct binding of VAC to EGFR. One should bear in mind that it is interesting to know whether the Lys-879, Ile-918, and Ala-920 mutations of EGFR prevent the benefits of VAC in DN mice, which deserves in-depth study. In addition, our results revealed that VAC inhibited the phosphorylation of the Y845 and Y1173 sites of EGFR in the context of DN, but the Y845 and Y1173 sites of EGFR did not bind to VAC according to the results of the molecular docking model. We speculated that VAC may bind to the Lys-879, Ile-918, and Ala-920 residues of EGFR, causing conformational changes in EGFR, exposing the Y845 and Y1173 sites, which are activated by their ligand EGF, thereby inducing phosphorylation of Y845 and Y1173. This hypothesis needs to be confirmed by more experimental evidence.

In conclusion, the results of this study revealed that administration with VAC effectively ameliorated renal damage by lowering blood glucose and restoring histological alterations in an experimental model of HFD/STZ-induced DN in mice. Moreover, we found that VAC ameliorated diabetes-induced renal injury by inactivating the EGFR/ERK1/2 signaling pathway.

## Supporting information

536FigS1-5-TabS1

## Supplementary Data

Supplementary data is available at *Acta Biochimica et Biophysica Sinica* online.
