# Supplementary material for: Vaccarin suppresses diabetic nephropathy through inhibiting the EGFR/ERK1/2 signaling pathway: Vaccarin ameliorates diabetic nephropathy
Source: Acta Biochim Biophys Sin (Shanghai). 2024 Aug 27;56(12):1860–74. doi: 10.3724/abbs.2024141 (PMC11972988; doi:10.3724/abbs.2024141)
Supplement: 536FigS1-5-TabS1 [file 536FigS1-5-TabS1.pdf]

A

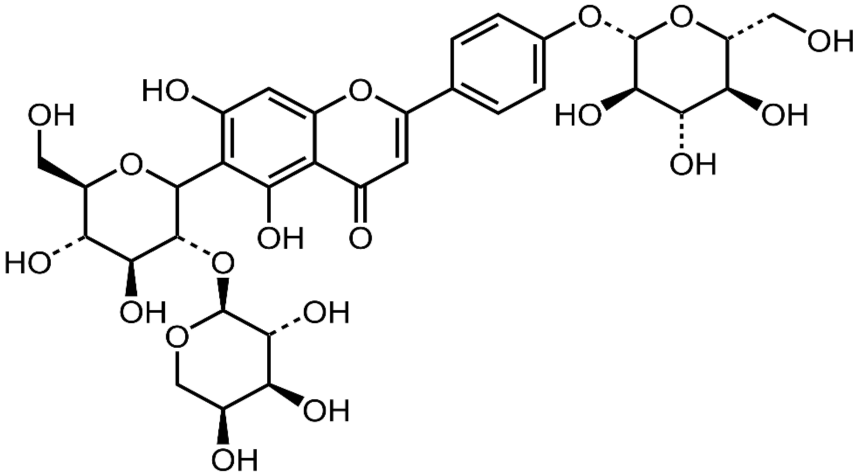

Supplementary Figure S1. (A) The chemical structure of VAC.

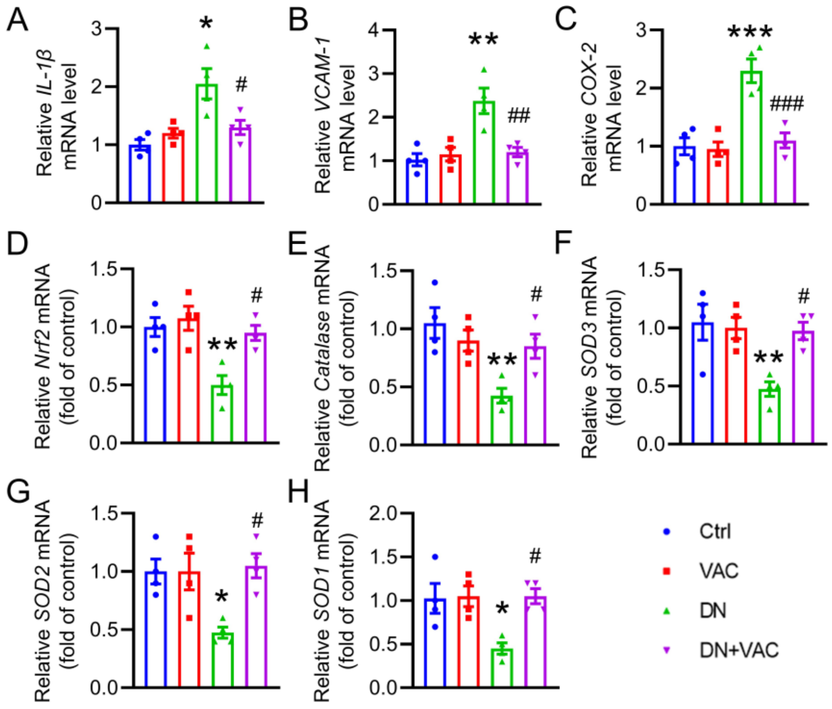

Supplementary Figure S2. VAC alleviated renal inflammation and oxidative burst in T2DM mice. (A-C) Relative mRNA levels of *IL-1β*, *VCAM-1* and *COX-2*. (D-H) Relative mRNA levels of *Nrf2*, *Catalase*, *SOD3*, *SOD2* and *SOD1*. $^{*}P < 0.05$, $^{**}P < 0.01$, $^{***}P < 0.001$ vs Ctrl. $^{\#}P < 0.05$, $^{\#\#}P < 0.01$, $^{\#\#\#}P < 0.001$ vs DN. $n = 4$.

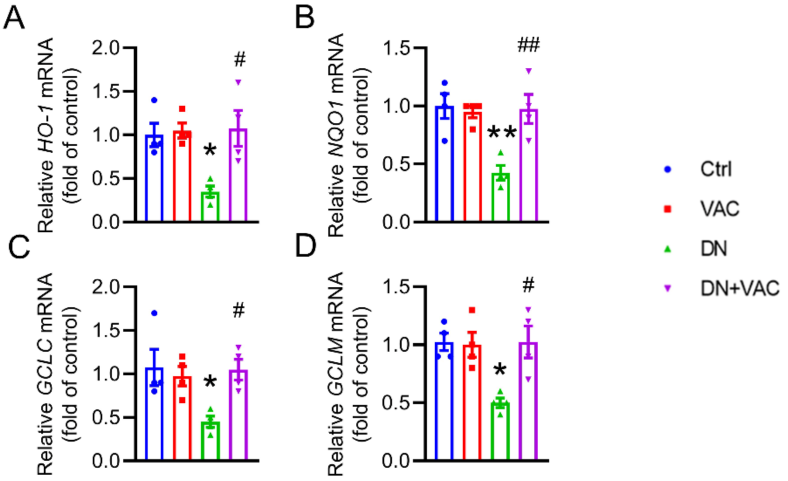

Supplementary Figure S3. The effect of VAC on the expression of Nrf2-dependent genes in T2DM mice. (A-D) RT-PCR analysis of *HO-1*, *NQO1*, *GCLC*, and *GCLM*. $^{*}P < 0.05$, $^{**}P < 0.01$ vs Ctrl. $^{\#}P < 0.05$, $^{\#\#}P < 0.01$ vs DN. $n = 4$.

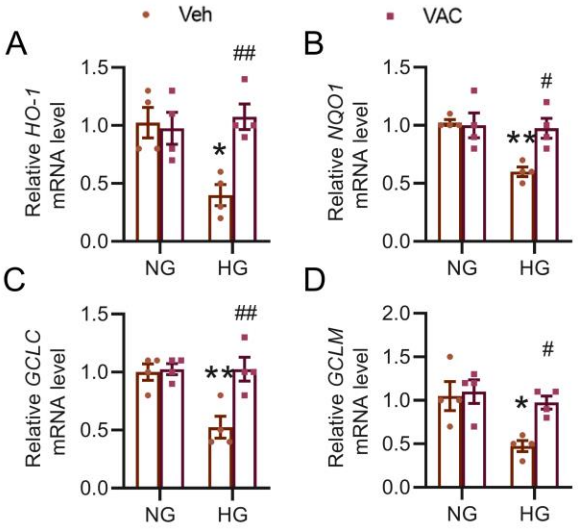

Supplementary Figure S4. The effect of VAC on the expression of Nrf2-dependent genes in HK-2 cells. (A-D) RT-PCR analysis of *HO-1*, *NQO1*, *GCLC*, and *GCLM*. $^{*}P < 0.05$, $^{**}P < 0.01$ vs NG. $^{\#}P < 0.05$, $^{\#\#}P < 0.01$ vs HG. $n = 4$.

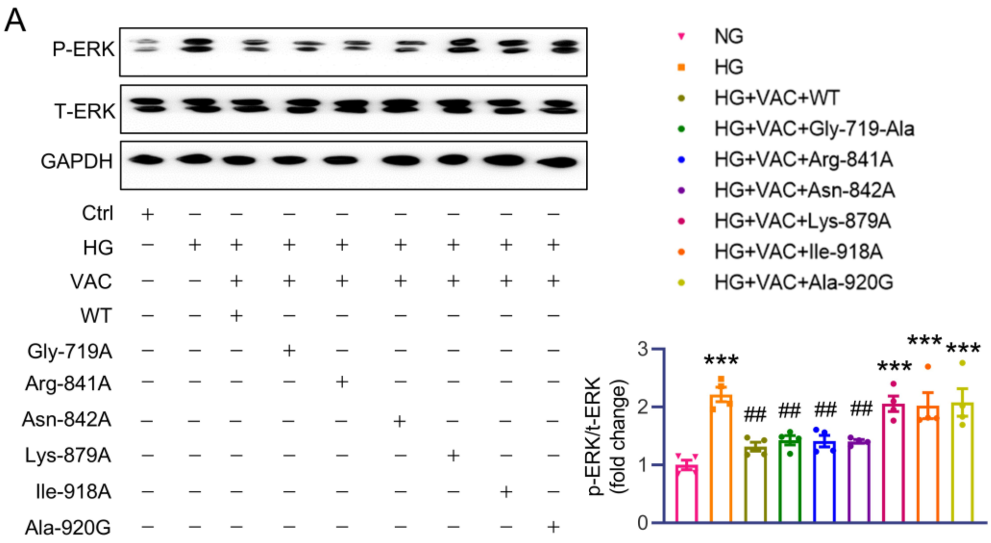

Supplementary Figure S5. Amino acid site mutations of EGFR were performed to confirm the interaction of VAC with EGFR

Supplementary Table S1. Primers for real-time quantitative PCR analysis.

Species	Gene	Sequence (5′→3′)
Mice	*β-actin*	F: ACCGCTCGTTGCCAATAGTGATG
		R: ACCGCTCGTTGCCAATAGTGATG
Mice	*Nrf2*	F: AGACGAGGCGGTACAAGTTT
		R: AAAAGGGCTCTGTGTTCCCA
Mice	*SOD2*	F: GCCCAAACCTATCGTGTCCA
		R: AGGGAACCCTAAATGCTGCC
Mice	*HO-1*	F: CATAGCCCGGAGCCTGAATC
		R: CTCAGCATTCTCGGCTTGGA
Mice	*NQO1*	F: AGATTAGGAGCCTCAGGGCA
		R: AAAGAGCTGGAGAGCCAACC
Mice	*GCLC*	F: GCTTTGGGTCGCAAGTAGGAA
		R: CTCCGATGCCGGATGTTTCT
Mice	*GCLM*	F: CTCGGGTGAGGTTTCTGCTT
		R: CCATCTTCAATCGGAGGCGA
Mice	*collagen* Ⅰ	F: TGGTCCTGCTGGTCCTGCTG
		R: CTGTCACCTTGTTCGCCTGTCTC
Mice	*TGF-β1*	F: GCAACAATTCCTGGCGTTACCTTG
		R: CAGCCACTGCCGTACAACTCC
Mice	*E-cadherin*	F: ATCCTGACCAGCAGTTCGTTGTTG
		R: GTTCCTCGTTCTCCACTCTCACATG

Mice	*α-SMA*	F: GCTGACAGAGGCACCACTGAAC
		R: AGTCACACCATCTCCAGAGTCCAG
Mice	*IL-1β*	F: TGGACCTTCCAGGATGAGGACA
		R: GTTCATCTCGGAGCCTGTAGTG
Mice	*VCAM1*	F: ATAGACAGCCCACTAAACGC
		R: TCAATGACGGGAGTAAAGGT
Mice	*COX-2*	F: TGAGCAACTATTCCAAACCAGC
		R: GCACGTAGTCTTCGATCACTATC
Mice	*p65-NFκB*	F: GCTCCTAAGGTGCTGACA
		R: ACCTCCGAAAGCGAGATA
Mice	*Podocin*	F: TCCTCTAGGGATGACAAGAAGGCA
		R: AATGGCAAAGGCAAAACCACA
Mice	*Nephrin*	F: CAGAAAAGCAGGGCACACAG
		R: CTGTACCTTGGGAAGCCTGG
Human	*β-actin*	F: CATGGAGTCCTGTGGCATCC
		R: CTCCTTCTGCATCCTGTCGG
Human	*Nrf2*	F: GTGCTGTCAAGGGACATGGA
		R: AGTGACTGAAACGTAGCCGAA
Human	*SOD2*	F: GCTGGAAGCCATCAAACGTG
		R: TGCTCCCACACATCAATCCC
Human	*HO-1*	F: TGTTGGAGCCACTCTGTTCC
		R: GCTCAAAAACCACCCCAACC

Human	*NQO1*	F: TTGAGCGAGTGTTCATAGGAGAG
		R: CCTTCTTACTCCGGAAGGGT
Human	*GCLC*	F: ACTTCATTTCCCAGTACCTTAACA
		R: GAAATCACTCCCCAGCGACA
Human	*GCLM*	F: AGCGAGGAGGAGTTTCCAGA
		R: TGACCGAATACCGCAGTAGC
Human	*Collagen Ⅰ*	F: CCACCAATCACCTGCGTACAGAAC
		R: TCACAGATCACGTCATCGCACAAC
Human	*TGF-β1*	F: AGCAACAATTCCTGGCGATACCTC
		R: TCAACCACTGCCGCACAACTC
Human	*E-cadherin*	F: ACAACGACCCAACCCAAGAATCTATC
		R: CAAGAGCAGCAGAATCAGAATTAGC
Human	*α-SMA*	F: CTCTGGACGCACAACTGGCATC
		R: CACGCTCAGCAGTAGTAACGAAGG
Human	*IL-1β*	F: TGAAAGCTCTCCACCTCCAGGGACA
		R: GAGGCCCAAGGCCACAGGTATTTTG
Human	*VCAM1*	F: CCTGCCATTGGAATGATAA
		R: TGCTTCTACAAGACTATATGAC
Human	*COX-2*	F: AACTCTGGCTAGACAGCGTAA
		R: AACCGTAGATGCTCAGGGAC
Human	*p65-NFκB*	F: ACTGCCGGGATGGCTTCTAT
		R: CCCACGCTGCTCTTCTTGGAA

Note. α-SMA: α-smooth muscle actin; TGF-β1: transforming growth factor beta 1
